# Inflammation, a Link between Obesity and Cardiovascular Disease

**DOI:** 10.1155/2010/535918

**Published:** 2010-08-05

**Authors:** Zhaoxia Wang, Tomohiro Nakayama

**Affiliations:** Division of Laboratory Medicine, Department of Pathology and Microbiology, Nihon University School of Medicine, 30-1 Ooyaguchi-kamimachi, Itabashi-ku, Tokyo 173-8610, Japan

## Abstract

Obesity, the most common nutritional disorder in industrialized countries, is associated with an increased mortality and morbidity of cardiovascular disease (CVD). Obesity is primarily considered to be a disorder of energy balance, and it has recently been suggested that some forms of obesity are associated with chronic low-grade inflammation. The present paper focuses on the current status of our knowledge regarding chronic inflammation, a link between obesity and CVDs, including heart diseases, vascular disease and atherosclerosis. The paper discusses the methods of body fat evaluation in humans, the endocrinology and distribution of adipose tissue in the genders, the pathophysiology of obesity, the relationship among obesity, inflammation, and CVD, and the adipose tissue-derived cytokines known to affect inflammation. Due to space limitations, this paper focuses on C-reactive protein, serum amyloid A, leptin, adiponectin, resistin, visfatin, chemerin, omentin, vaspin, apelin, and retinol binding protein 4 as adipokines.

## 1. Introduction

Obesity, the most common nutritional disorder in industrialized countries, is associated with an increased mortality and morbidity of cardiovascular disease (CVD) [1]. The World Health Organization estimates that more than 1 billion adults worldwide are overweight, 300 million of whom are clinically obese—defined as having a body mass index (BMI) equal to or greater than 30 kg m^−2^, or a waist circumference greater than 94 cm for men and 80 cm for women [2]. Obesity is a chronic, multifactorial, and complex disease resulting from a long-term positive energy balance, in which both genetic and environmental factors are involved [3, 4]. It was recently suggested that some forms of obesity are associated with chronic low-grade inflammation [5].

CVDs, including heart disease, vascular disease and atherosclerosis, are the most critical global health threat, contributing to more than one-third of the global morbidity. In most cases, these clinical conditions result from atherosclerosis, which was once identified as a lipid-storage disease. At the present time, CVD is recognized as a chronic inflammatory condition of the vessel wall that results from the transendothelial passage (transcytosis) of cholesterol-rich atherogenic Apo-B lipoproteins (VLDL, IDL and LDL) from the plasma into the intima. These lipoproteins are retained in the subendothelial space, which leads to infiltration of macrophages and T cells that ultimately then interact with each other and with the cells of the arterial wall [6, 7]. It is likely that inflammation induced by obesity accelerates the atherosclerosis. Adipose tissue is recognized as an important player in obesity-mediated CVD. In 1994, adipose tissue was first identified as the source of the hormone leptin, opening the door for a new era of research that focused on adipocyte endocrinology [8]. It is now apparent that adipocytes have a more complex physiological role [9]. Adipocytes produce large numbers of hormones, peptides, and other molecules that affect cardiovascular function, not only in an endocrine manner, but also by autocrine and paracrine mechanisms [10]. This might lead to cytokine-mediated inflammatory, changes in the liver, systemic inflammation and atherosclerosis.

This paper focuses on the inflammation related to obesity and CVD. It will discuss the methods of body fat evaluation in humans, the endocrinology and distribution of adipose tissue in the genders, the pathophysiology of obesity, the relationship among obesity, inflammation and CVD, and the adipose tissue-derived cytokines known to affect inflammation.

## 2. Methods of Evaluation of Body Fat in Humans

There are many methods that can be used to evaluate body fat in different populations [2, 11, 12]. While anthropometric measurements of weight-for-height have been traditionally used to evaluate obesity, more recently, BMI has become a standard parameter. BMI is defined as weight in kilograms divided by height in square meters. The normal range is 19–24.9 kg/m^2^, with overweight defined as 25–29.9 kg/m^2^, and obesity as ≥30 kg/m^2^. BMI is not always a reliable measurement of body composition in individuals, particularly in older and younger people. Unfortunately, BMI does not provide any insight into regional body fat distribution. Thus, simple anthropometric measurements, such as waist circumference, can also be used to determine the valid index of visceral fat accumulation, in addition to being able to serve as an indicator of health risks associated with visceral obesity. A waist circumference of greater than 102 cm in men and 88 cm in women is a risk factor for CVD. A particularly important anthropometric parameter that has been increasingly applied in recent years is sagittal abdominal diameter (SAD) [13]. Using a simple caliper that was originally developed by Kahn, this anthropometric indicator can measure visceral fat tissue alone [14].

With regard to other techniques, one of the first that should be considered is the measuring of body density, as this provides information on the relationship between the body mass and volume. With tetrapolar bioelectric impedance analysis, data is obtained by measuring the resistance of the body exposed to the impact of an alternating current of 50 kHz at a strength of 800 *μ*A. Radioisotopic techniques use deuterium or tritium as markers to measure the total body liquid and total body potassium. Infrared spectrometry is a simple but not particularly reliable method, based on the application of two sources of monochromatic light. Ultrasonographic measuring of fat tissue is currently the favored technique by which one can measure both the subcutaneous and visceral fat tissues. Measurements are carried out using a 7.5- and 3.5-mHz transducer for the subcutaneous and visceral fat tissue, respectively. The most accurate method for measuring central obesity is through the use of magnetic resonance imaging or computer-assisted tomographic scanning. Unfortunately, these approaches are too expensive for routine use.

## 3. Endocrinology and Distribution of Adipose Tissue between Genders

**Figure 1:**
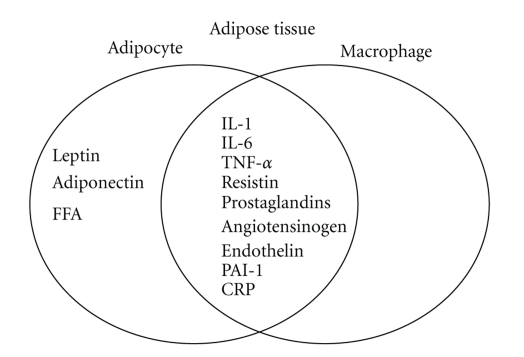
Cytokines secreted by adipocytes and/or macrophages in human adipose tissue.

It is now apparent that adipose tissue is not simply a storage reservoir of fat, but is an active endocrine organ that plays multiple roles in the body. Adipose tissue contributes to the inflammatory process in obese subjects in both vascular and nonvascular tissues [15]. Abnormal levels of metabolites, such as lipids, fatty acids and, cytokines from adipose tissue, activate monocytes and increase the secretion of inflammatory cytokines. Adipose tissue from obese individuals contains activated macrophages that together with adipocytes produce various cytokines (Figure 1). These include inflammation-related adipokines such as leptin, adiponectin, tumor necrosis factor alpha (TNF-*α*), interleukin-1 (IL-1), interleukin-6 (IL-6), procoagulant substances such as PAI-1, vasoactive substances such as leptin, angiotensinogen and endothelin, and molecules that may contribute to insulin resistance such FFA, TNF-*α* and resistin. IL-1 signaling involves the type I Interleukin 1 receptor (IL-1R/IL-1R1), a Toll-like receptor that heterodimerizes with the IL-1R accessory protein (IL-1RAcP). Interleukin 1 receptor antagonist (IL-1Ra) is an anti-inflammatory cytokine that binds to IL-1R in competition with the proinflammatory cytokine IL-1. The relative occupancy of the IL-1R1-IL-1RAcP receptor complex with IL-1 agonist or with IL-1Ra determines whether the inflammatory signaling is “on” or “silenced”, respectively. IL-1*β* induction of IL-6 and prostaglandin E2 (PGE2) signaling is indicated in Figure 3. In obesity, these cytokines are released into the circulation by adipose tissue, stimulating hepatic CRP production. Levels of the prothrombotic molecule PAI-1 are also increased, whereas adiponectin, which is produced exclusively by adipocytes, is decreased in obesity. One of the key vasoactive substances produced by adipocytes is leptin, which is an important regulator of food intake. Other adipocyte-derived molecules, including prostaglandins, adiponectin, and the more recently discovered resistin, affect metabolic function and might play a role in causing cardiovascular end-organ damage.

Serum adipokine levels are elevated in humans and animals with excess adiposity. Visceral fat appears to produce several adipokines more actively than subcutaneous adipose tissue, and an increased abdominal adiposity in the visceral depot renders these individuals more prone to metabolic and cardiovascular problems [16]. Health problems associated with obesity are generally related more to the central (abdominal, visceral) distribution of fat rather than to the amount of fat, and the distribution of fat differs between males and females [17, 18]. Men exhibit a more central accumulation of fat, whereas women exhibit a more gluteal/femoral accumulation. The original definition of obesity for males (android type) and females (gynoid type) dates to the first clinical observations made by Vague in 1947. The greatest health risk is associated with fat distribution in the central or upper body (android) parts. Recent research [19, 20] has shown that sex hormones play an important role in obesity and that there are differences in the occurrence of insulin resistance and heart diseases that are dependent upon gender. Findings have indicated that both the total amount of fat that an individual carries and the distribution of that fat are important. At present, it is difficult to accurately measure fat in the body, and there is currently no simple method available for routine clinical use.

Epidemiological and clinical evidence strongly suggests a major role for sex steroid hormones in the regulation of adipose tissue distribution. Sex steroid hormones, such as estrogen, progesterone, and androgen, are involved in the metabolism, accumulation, and distribution of adipose tissues. Normal distribution of body fat occurs when sex steroid hormones are present. If a decrease in sex steroid hormones occurs, such as that seen during aging or gonadectomy, there is a greater tendency for obesity states, in addition to increases in major risk factors for CVD.

## 4. Mechanisms of the Relationship amongObesity, Inflammation, and CVD

### 4.1. Systemic Inflammation

**Figure 2:**
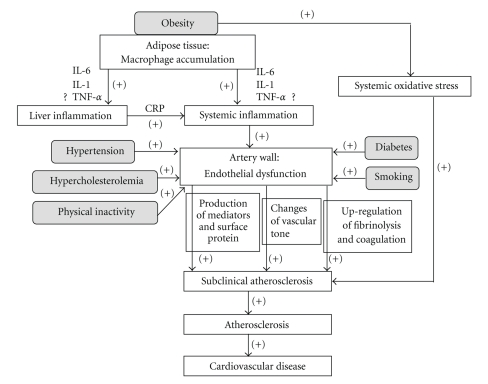
Mechanism of the relationship between inflammation induced by obesity and cardiovascular disease. Gray box shows the traditional cardiovascular disease (CVD) risk factors. The arrow and plus symbols indicate the enhanced courses. Smoking, obesity, hypertension, diabetes, physical inactivity and hypercholesterolemia are established risk factors of CVD. In obese individuals, macrophages first accumulate within the adipose tissue, leading to local inflammation. As the obesity increases, several proinflammatory factors, including IL-1, IL-6 and TNF-*α*, are produced in the adipose tissue. Macrophage accumulation and the subsequent local inflammation are believed to result in numerous metabolic dysfunctions that typically accompany obesity, including systemic inflammation. Endothelial dysfunction occurs during the early stages of atherosclerosis and is responsible for the pathophysiological changes in subclinical atherosclerosis, which include changes in a variety of mediators, surface proteins, and in autacoids that are involved in vasomotion, coagulation and inflammation. Obesity also can increase systemic oxidative stress independently of blood glucose and diabetes. One of the major events of atherosclerosis is CVD.

**Figure 3:**
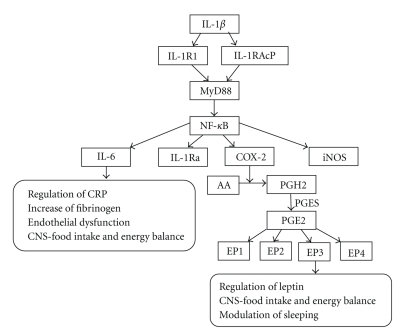
Interleukin-1*β* (IL-1*β*) induction of interleukin-6 (IL-6) and prostaglandin E2 (PGE2) signaling. IL-1*β* binds to the IL-1R1/IL-1R1AcP heterodimer, which then initiates the signaling cascade that causes the translocation of the transcription factor nuclear factor-*κ*B (NF-*κ*B) into the nucleus, where it induces the transcription of pro- and anti-inflammatory genes including inducible nitric oxide synthetase (iNOS), IL-6, IL-1Ra and cyclooxygenase-2 (COX-2). COX-2 catalyses the conversion of arachidonic acid (AA) to prostaglandin H2 (PGH2). PGH2 is converted into PGE2 by terminal PGE synthase (PGES). PGE2 signals occur via four different G-protein coupled receptors, EP1R-EP4R, each of which has multiple splice variants with different signaling properties.

As individuals become obese and their adipocytes enlarge, the adipose tissue undergoes molecular and cellular alterations that subsequently affect systemic metabolism (Figure 2). First, macrophages accumulate within adipose tissue, leading to local inflammation. Several proinflammatory factors are produced in adipose tissue as obesity increases. When compared to lean individuals, adipose tissue in obese individuals shows higher expression of proinflammatory proteins, including TNF-*α* and IL-6 [21, 22]. Macrophage numbers in adipose tissue also increase with obesity [23], apparently acting as scavengers of apoptotic adipocytes. It also has been reported that there is a marked increase in these scavengers in obese subjects [24]. Macrophage accumulation and the subsequent local inflammation are believed to result in numerous metabolic dysfunctions that accompany obesity, including systemic inflammation and atherosclerosis.

Visceral fat secretes more cytokines than subcutaneous adipose tissue [16]. A recent study elegantly demonstrated that transplantation of visceral adipose tissue from genetically obese mice into Apoe-deficient mice increased atherosclerosis in the recipient animals, suggesting that inflamed adipose tissue exerts distinct vascular effects, presumably through inflammatory cells such as macrophages within the visceral adipose tissue [25]. Macrophages within visceral adipose tissue are known to express and release cytokines. These cytokines reach the liver though the portal circulation, where they can stimulate hepatic inflammation [26], thereby inducing a chronic systemic inflammatory response.

### 4.2. Endothelial Dysfunction

Clinical and experimental data support a link between systemic inflammation and endothelial dysfunction. Mounting evidence shows that disturbed endothelial function may be an early marker of an ongoing atherosclerotic process. Thus, endothelial dysfunction has increasingly been recognized to play an important role in a number of conditions associated with a high prevalence of atherosclerotic CVD. Inflammatory cytokines are important protagonists in the formation of atherosclerotic plaques, eliciting effects throughout the atherosclerotic vessel. Importantly, the development of atherosclerotic lesions, regardless of risk factors (e.g., diabetes, hypertension, obesity), is characterized by the disruption of the normal function of endothelial cells.

The reasons for coronary endothelial dysfunction are complex and may involve ischemia/reperfusion injury. Smoking, obesity, hypertension, diabetes, physical inactivity, and hypercholesterolemia are established atherogenic risk factors. Endothelial dysfunction is regarded as an early stage of atherosclerosis, which is a chronic inflammatory disease [27]. Chronic inflammation is a major contributing factor to atherosclerosis and various markers of inflammation, fibrinolysis, and coagulation are upregulated in patients with established atherosclerotic disease. For vascular homeostasis, endothelial cells are of the utmost importance and they produce a variety of mediators, surface proteins, and autacoids involved in vasomotion, coagulation, and inflammation. Adipose tissue expresses enzymes involved in the angiotensin system (RAS) (renin, angiotensin-converting enzyme (ACE)), as well as the nonrenin-angiotensin system (NRAS) (cathepsin D, cathepsin G, tonin, chymase) [28]. The identification of elevated CRP as a transient independent risk factor for endothelial dysfunction might provide an important clue for linking a systemic marker of inflammation to the progression of atherosclerotic disease. Thus, CRP has been proposed for risk assessment of CVD in the at-risk general population. Available evidence suggests that low-grade inflammation is accompanied by decreased bioavailability of endogenous NO and that TNF-*α* may play a key role in these events. The adipose tissue constitutes a source of other vasoactive factors, such as leptin, serum amyloid A (SAA), or apelin, among others [29]. Since blood vessels express receptors for most of the adipocyte-derived factors, adipose tissue seems to play a key role in cardiovascular physiology via the existence of a network of local and systemic signals. Therefore, these data demonstrate that markers of inflammation have independent predictive value for clinical and subclinical CVD beyond that of the traditional risk factors.

### 4.3. Subclinical Atherosclerosis and CVD

The development of atherosclerosis in obesity stems from a constellation of interrelated proatherogenic mechanisms. It is well established that a higher BMI is associated with subclinical inflammation, as reflected by increased CRP levels [30], and increased systemic oxidative stress that is independent of blood glucose and diabetes [31]. Recent evidence has suggested that leptin stimulates cholesterol uptake by macrophages, particularly in the presence of high glucose. This then triggers the formation of foam cells and the development of atheromatic lesions. Obesity-related hypoadiponectinemia might also contribute to impaired endothelial function, increased vascular ROS production and overall proatherogenic effects [32]. Finally, increased release of proinflammatory cytokines by adipose tissues, including IL-6, IL-1, and TNF-*α*, sustains vascular wall inflammation and promotes pro-atherogenic gene expression [33].

There is interest in identifying markers of subclinical atherosclerosis, such as coronary artery calcium (CAC) and carotid intimal medial thickness (CIMT), in order to facilitate an earlier diagnosis and possible prevention of CVD. CRP levels were found to be correlated with CIMT in a group of young subjects [34], but not in older individuals [35]. In other studies, levels of IL-6 have been shown to be associated with the amount of CAC [36], and the CD40 ligand, which is a marker of enhanced innate immunity, has been found to be correlated with CIMT in human subjects [37]. Since leptin levels have been shown to be associated with CAC independently of body weight measures or other risk factors, this points to a possible proatherogenic role for leptin [38].

## 5. Adipose Tissue-Derived Cytokines Known to Affect Inflammation

### 5.1. CRP

Of the many positive and negative acute-phase reactants, perhaps the most recognized is CRP, which is a member of the pentraxin family that attaches to the plasma membrane of damaged cells causing cell death through activation of the complement cascade [39]. More than 20 prospective epidemiologic studies have demonstrated that high-sensitivity CRP is an independent predictor of myocardial infarction, stroke, peripheral arterial disease, and sudden cardiac death, even in apparently healthy individuals [40, 41]. Clearly, CRP is one of the strongest markers of chronic inflammation, and it has been reported that it also directly participates in the coronary and aortic atherosclerosis that leads to cardiac events [42].

Ouchi et al. [43] confirmed CRP mRNA expression in human adipose tissue using quantitative real-time polymerase chain reaction. In the same article, the authors proposed that adipose tissue is an important source of circulating CRP. However, they made no attempt to investigate the stimuli able to induce CRP. Esposito et al. [44] investigated the effects of weight loss and lifestyle changes on vascular inflammatory markers in obese women. After 2 years, they found that BMI, as well as the serum concentrations of IL-6, IL-18, and CRP, decreased more in the intervention group than in the control subjects, whereas the adiponectin levels significantly increased. The beneficial effects of a Mediterranean-style diet on endothelial function and vascular inflammatory markers have been documented in patients with metabolic syndrome. When compared to patients consuming a control diet, patients consuming a Mediterranean-style diet have significantly lower serum concentrations of high-sensitivity CRP, IL-6, IL-7, and IL-18 as well as a decreased insulin resistance [45]. In a quartile analysis of the percent weight reduction, the largest weight reduction quartile did not show significant decreases in the CRP levels, whereas the middle quartiles showed remarkable CRP decreases. Based on inflammatory status, there may be an optimal pace of exercise combined with weight loss [46]. Two recent studies have demonstrated that exercise training in conjunction with weight reduction significantly affected the CRP levels, body composition, and human left ventricular growth [47, 48].

### 5.2. Serum Amyloid A (SAA)

Serum amyloid A (SAA), an important marker of inflammation, is an apolipoprotein that is mainly synthesized in mammalian liver [49]. Human SAA is a 12.5-kDa protein whose levels can increase up to 1,000-fold in the serum 24–36 h after infection or injury, decline after 4–5 days, and then return to baseline after 10–14 days [50]. The human genome encompasses four SAA genes, of which three encode functional proteins. SAA1 and SAA2 are highly homologous reactants whose concentration can increase upon infection, trauma, and obesity [51, 52], whereas SAA3 is a pseudogene and SAA4 is a constitutively expressed minor constituent of the nonacute-phase HDL [53].

SAA has proven to be a suitable and sensitive indicator of the various stages of inflammation involved in inflammatory disorders. SAA is comparable to CRP, as both are major acute phase proteins that can increase up to 1,000-fold and reach 1 mg/mL in the serum under stimulation [50]. They can be produced by the liver under inflammatory stimuli, and their effects are mediated through pro-inflammatory cytokines (IL-1 and TNF-*α*) and “messenger” cytokines (IL-6) [54]. However, in contrast to CRP, which is mainly expressed in the human liver, SAA is expressed in both the liver and adipose tissue. SAA is now accepted as an adipokine that is produced by adipocytes and which directly mediates obesity-associated inflammation. Hence, SAA might serve as a better indicator of obesity and obesity-associated diseases, especially when vascular diseases and metabolic disorders are present.

SAA is known to be a marker for obesity, as its expression is well correlated with obesity [55]. Some studies have shown that SAA levels are positively associated with BMI levels and that weight loss led to decreased SAA levels. In 1999, Danesh et al. [56] first reported that concentrations of SAA protein were strongly correlated with obesity. Since then, more than ten studies have shown that SAA is strongly associated with obesity [57–59]. In addition, it has been shown that SAA gene expression is increased in the adipose tissue of obese subjects and is significantly correlated with adipocyte size and inflammatory biomarkers [52].

Recent studies have shown that SAA elevation can predict cardiovascular events analogously with or even better than CRP by itself [60–62] and in this sense, it has been speculated that SAA might be one of the links or even a proatherogenic risk factor between inflammation and CVD [63, 64]. SAA is able to both alter vascular proteoglycans in a proatherogenic manner [65] and stimulate the production of various inflammatory mediators in cultured vascular endothelial cells, neutrophils, and monocytes [66]. Endothelial cells, smooth muscle cells, monocytes, and macrophages in atherosclerotic lesions have been reported to account for the extrahepatic production of SAA, as the presence of both SAA mRNA and protein products have been detected in these cell types [67]. SAA has also been accepted as being a biomarker of cerebrovascular disease and carotid artery intima-media thickness, which is an early index of atherosclerosis [68–71]. However, a very recent study indicated that SAA does not mediate early atherosclerosis [57].

SAA has also been found to be associated with metabolic disorders, such as diabetes, insulin resistance, and metabolic syndrome [72, 73]. Additionally, genes critical for insulin sensitivity were also found to be downregulated in adipocytes treated with recombinant SAA [74].

### 5.3. Leptin

Leptin, which was the first adipocyte hormone identified, influences food intake through direct effects on the hypothalamus [75]. The adipocyte-derived hormone leptin has actions in the brain (e.g., hypothalamus, cortex and limbic areas) and in a number of peripheral tissues as well (e.g., cells of the pancreas, liver and immune system). However, the central action of leptin in the brain, and in particular the hypothalamus, has been best characterized with regards to energy homeostasis and its importance for reproductive functions [76]. Moreover, disruption of peripheral leptin signaling in mice has been shown to cause no significant changes in either the energy balance or glucose homeostasis [77].

Mice lacking the gene coding for leptin (named *ob/ob* mice) are obese and diabetic. When *ob/ob* mice are treated with regular injections of leptin, they show reduced food intake, increased metabolic rate, and weight loss [78]. These effects appear to be mediated mainly by the central nervous system, as intracerebroventricular injection of leptin produces significant effects at much lower doses than those required by systemic injection. Systemic injections of leptin have a beneficial effect in children with congenital leptin deficiencies [79]. In a pioneering study, administration of exogenous leptin to individuals with lipoatrophic diabetes resulted in marked reductions in triacylglycerol concentrations, liver volume, and glycated hemoglobin. Ultimately, this treatment resulted in the discontinuation of or large reductions in the patient's antidiabetic therapy [80]. Unfortunately, leptin concentrations are already high in most obese individuals because of the increased amount of leptin-secreting adipose tissue [81]. In these individuals, increasing the leptin concentrations only induces the target cells to become resistant to actions of the hormone. Therefore, further studies need to be undertaken to clarify potential therapeutic strategies using leptin in these types of patients.

Leptin is involved in the control of not only energy homeostasis but also immunity. During fasting/starvation, when plasma leptin levels decline, neural pathways in the hypothalamus cause the appetite to increase and energy expenditure to decrease as the body attempts to restore its fat stores [82]. In addition, the fall in plasma leptin diminishes thyroid hormone production and inhibits the reproductive axis, both of which help to save energy during nutritionally lean times [83]. These metabolic effects of leptin are in part centrally mediated by activation of the hypothalamic-sympathetic nervous system axis [84]. In addition to the complete leptin deficiency disorder, relative leptin deficiency is an emerging clinical syndrome that is now being seen more often in several clinical conditions, including congential or acquired lipodystrophy as well as exercise-induced energy deficiency and hypothalamic amenorrhea or anorexia nervosa. Leptin replacement therapy might prove to be a therapeutic option for patients with these disorders [85]. Very recently, administration of chemical chaperones that decrease ER stress also restored leptin sensitivity in diet-induced obese mice [86]. In obese subjects who have lost weight, modifications that lead to decreased energy expenditure may predispose the individual to regain the weight. However, when subjects are administered “replacement” doses of leptin that restore their circulating leptin concentrations to preweight-loss levels, the weight gain can be prevented [87]. This suggests that the weight-reduced state is a condition of relative leptin deficiency. Recent reports have shown that in addition to its action on the hypothalamus, leptin may also act on the cortex and limbic areas, which are involved in cognitive and emotional regulation of feeding behavior [88]. Teleologically, the adaptations mediated by reduced leptin may have evolved as a protection against the threat of starvation by limiting energy use and enhancing energy storage [89].

The potential effects of leptin in the pathophysiology of cardiovascular complications of obesity remain diverse [90]. While some studies [91, 92] have indicated that circulating leptin levels are not significantly related to the risk of CVD or mortality in a diabetic population, these studies did find that leptin was associated with obesity and inflammatory markers. Even so, other reports have suggested that leptin does contribute to atherosclerosis and CVD in obese subjects [93]. Therefore, this protein may elevate the blood pressure by stimulating the autonomic nervous system. Leptin has been found to have multiple effects on the cells of the artery wall. In human vascular endothelial cells, leptin upregulates the expression of the plasminogen activator inhibitor-1 [94], and leptin also helps modulate ACAT1 expression and cholesterol efflux from human macrophages [95]. In addition, leptin has been reported to increase nitric oxide (NO) bioavailability in blood vessels via the activation of endothelial NO synthase (eNOS) [96] and inducible NO synthase (iNOS) [97] in the endothelial and smooth muscle cells, respectively. Recent studies that measured coronary artery disease have demonstrated that hyperleptinemia was associated with coronary atherosclerosis [98, 99], with the association determined to be independent of insulin resistance. Other studies have shown that leptin may have a role in neointimal formation in response to arterial injury [100, 101]. In fact, very obese, leptin-deficient mice have been found to be protected from atherosclerosis despite all of the metabolic risk factors, suggesting that this hormone may directly contribute to the risk of vascular disease [102]. Moreover, in a prospective study in humans in which anthropometric and metabolic risk factors were controlled, the circulating leptin concentrations were shown to be an independent risk factor for predicting cardiovascular events [103]. Therefore, when chronically elevated concentrations of leptin are seen in obese individuals, this may indicate a predisposition to progression of atherosclerosis in these individuals.

### 5.4. Adiponectin

Adiponectin is a product of adipocytes, and its levels in humans decrease in obese subjects [104]. As one of the most extensively studied adipokines, adiponectin has 3 different oligomers, each of which may have a different biological function [105]. The major receptors for adiponectin are AdipoR1 and AdipoR2. These belong to a new family of receptors that are predicted to contain seven transmembrane domains but which will be structurally and functionally distinct from the G-protein coupled receptors. A recent study has shown that AdipoR2 stimulates energy dissipation by increasing fatty acid oxidation while inhibiting oxidative stress and inflammation [106, 107]. Adipocytes secrete high levels of adiponectin that then exert anti-inflammatory effects, most notably in atherosclerotic plaques [108]. These effects occur due to the suppression of TNF-*α* and proinflammatory cytokines such as IL-6 and interferon-c, along with the induction of other anti-inflammatory factors such as the IL-1 receptor antagonist [109]. In contrast, adiponectin levels have been shown to be low in several different forms of insulin resistance. In vivo, adiponectin enhances energy consumption and fatty acid oxidation in the liver and muscle, which reduces the tissue triglyceride content, thereby further enhancing the insulin sensitivity [110]. In adiponectin transgenic mice, there is improvement of the lipid profile [111, 112], and when plasma triglycerides are reduced, this leads to an increased VLDL catabolism in the skeletal muscle [113]. Taken together with its metabolic and anti-inflammatory effects, it has been proposed that adiponectin not only contributes to the beneficial effects of body weight loss but also has a role in modulating the cardiovascular system.

As might be expected based on the above observations, adiponectin promotes an antiatherogenic and anti-inflammatory program of gene expression and function in the vessel wall. Adiponectin downregulates the expression of adhesion molecules on the endothelial cells and directly improves endothelial dysfunction [114, 115]. Adiponectin also reduces proliferation in a receptor-independent fashion in the vascular smooth muscle cells [116]. In a very recent study, it has been shown that adiponectin reduces lipid accumulation, down-regulates the expression of scavenger receptors in macrophages, and promotes macrophage polarization, all of which play a role in anti-inflammatory activities [117]. Other studies have also indicated that adiponectin has an important role in cardiovascular protection. Hypoadiponectinemia is found in patients with angiographically demonstrated coronary artery disease [118]. In obese children, it has been reported that reduced adiponectin concentrations are of more importance than conventional cardiovascular risk factors, and that this inflammation status is related to early atherosclerosis [119]. However, in a large prospective study that was combined with a meta-analysis of previously published prospective studies, the adiponectin levels at baseline were found to be rather weak predictors of CVD risk [120]. However, other studies have shown that adiponectin exerts beneficial effects at nearly all stages of the atherogenesis process [121], and that the adiponectin levels are inversely correlated to the progression of the coronary artery calcium in both diabetic and nondiabetic subjects [122]. Serum total and high-molecular weight adiponectin are also associated with biomarkers of inflammation, insulin resistance, and endothelial function, all of which are independent risk factors of CVD [123].

### 5.5. Resistin

Resistin, which is one of the most recently identified adipokines, has been proposed to be an inflammatory marker for atherosclerosis. *While it has been shown to induce increases in CRP production by the macrophages [124], resistin is an example of the new adipokines that appear to have contrasting roles when examined in mice versus humans.* For example, confirmation of the results found in mice has proven to be difficult in human populations. This may be due to the fact that resistin appears to be derived from different sources in humans as compared to rodents. This protein was initially shown to be released in large amounts from mouse adipocytes, with obese mice having elevated levels that were accompanied by insulin resistance [125]. However, investigations in humans suggest that resistin is expressed in adipocytes with monocytes and macrophages [126, 127]. This lack of homology between the human and mouse resistin genes suggests a potential divergence in function [128]. Since macrophages are known inflammatory modulators, resistin may be an inflammatory marker in humans. Supporting this possible inflammatory role in humans are results that show recombinant resistin activates human endothelial cells, as measured by an increased expression of endothelin-1 and various adhesion molecules and chemokines, while simultaneously increasing the CD40-ligand signaling by down-regulating the tumor necrosis factor receptor-associated factor-3 [129]. Moreover, Calabro et al. [130] has shown that resistin can promote human coronary artery smooth muscle cell proliferation by activation of the extracellular signal-regulated kinase 1/2 (ERK) and phosphatidylinositol 3-kinase (PI3 K) pathways. Taken together, these findings suggest a possible mechanistic link between resistin and cardiovascular disease via proinflammatory pathways.

In addition, there have been many recent reports that support a role for resistin in obese rodent models. Resistin has been found to modulate nutritional regulation and may possibly play a role in maintaining fasting blood glucose levels [131]. Further rodent studies have also suggested that resistin mRNA levels are higher in abdominal fat depots, as compared to deposits in the thigh [132], and that these serum resistin levels are positively correlated with BMI [133]. Recent investigations in humans have shown there are higher serum resistin levels in obese subjects as compared to lean subjects. These higher levels were also positively correlated with changes in the BMI and the visceral fat area [134, 135]. Lee et al. [136] found higher circulating resistin levels in obese mice when compared to their lean counterparts. Additional studies have reported significant reductions in circulating resistin levels following moderate weight loss [137] and postgastric bypass [138]. Collectively, these observations suggest that resistin may indirectly be involved in the nutritional regulation in humans.

### 5.6. Visfatin

Visfatin, also known as nicotinamide phosphoribosyltransferase (NAMPT), which was previously known as a pre-B cell colony-enhancing factor (PBEF), functions as a growth factor for early B cells within the immune system [139]. Fukuhara et al. [140] demonstrated that visfatin is a secreted protein that is expressed and regulated by the adipose tissue. As compared to subcutaneous adipose tissue, there are greater amounts of visfatin within visceral fat depots. Furthermore, this study indicated that visfatin could bind to and activate insulin receptors, similar to that seen for insulin both in vivo and in vitro. However, this effect of visfatin is controversial. For example, Revollo et al. were unable to reproduce the insulin-mimetic activity of this protein, even though a significant physiological role in the regulation of beta-cell function through the NAD biosynthetic activity was detected. Thus, the authors suggested that NAMPT could play an important role in the control of glucose metabolism [141]. After these novel findings, Fukuhara et al. decided to retract their previously published paper [142].

The visfatin peptide was originally discovered in the liver, skeletal muscle, and bone marrow and found to act as a growth factor for B-lymphocyte precursors. This peptide is not only produced by white adipose tissue (WAT), but also by endotoxin-challenged neutrophils, and is able to prevent apoptosis via a mechanism mediated by caspases 3 and 8 [143]. Circulating visfatin levels are closely correlated with WAT accumulation and visfatin mRNA levels increase in the course of adipocyte differentiation. Visfatin expression is up-regulated by IL-6 and TNF-*α*, and is down-regulated by GH [144]. Insulin has no effect on visfatin mRNA [145]. Moreover, visfatin is up-regulated by the peroxisomal proliferator-activated receptor (PPAR)-alpha and PPAR-gamma agonists in obese rats. Since it has been shown to be associated with improved glycemic control and lipid profiles, this suggests that PPAR-alpha and PPAR-gamma agonists may act, at least in part, via the up-regulation of visfatin expression [146]. In addition to inducing chemotaxis and the production of IL-1, TNF-*α*, IL-6, and costimulatory molecules by CD14C monocytes, visfatin also increases their ability to induce alloproliferative responses in lymphocytes, effects which are mediated intracellularly by p38 and MEK1 [144].

Possible associations between circulating visfatin and anthropometric or metabolic parameters in obesity and type 2 diabetes have been found in some but not all reported studies [147–149]. These contradictory findings may be due in part to the considerable differences found in the visfatin immunoassays [150]. In human studies, it has been shown there is a positive correlation between the visceral adipose tissue visfatin gene expression and BMI, along with a negative correlation between BMI and subcutaneous fat visfatin [151]. This suggests that visfatin regulation varies within different depots and that the adipose depot ratios are highly dependent upon the obesity of the subjects. A wide population study in humans has recently discovered a direct correlation between plasma visfatin and the BMI and body fat content in males only. This study failed to find any differences in the expression between the visceral and subcutaneous fat depots [152].

Several studies have shown that there are different disorders that exhibit altered plasma levels of this protein [153–156]. Thus, visfatin plasma concentrations may potentially be related to lipid metabolism [157] and the inflammatory response [158]. Since an increased expression of this protein has been observed in the macrophages of unstable carotid and coronary atherosclerosis in humans [159], and there is a negative association between the visfatin plasma levels of visfatin and vascular endothelial function [160], it has been proposed that visfatin plays a role in plaque destabilization. NAMPT, which was originally identified as PBEF, has been shown to act as a cytokine independent of its enzymatic activity, and thus plays a major part in regulating immune responses [161]. Since NAMPT has been implicated in the pathogenesis of several acute or chronic inflammatory conditions, such as atherosclerosis and CVD [161], it may act as a pro-inflammatory cytokine and potentially have a beneficial effect on insulin secretion.

At the present time, the role of visfatin in the modulation of glucose metabolism, as well as its ability to bind to the insulin receptor is still under debate [162–164]. As a number of inconsistencies among the different visfatin studies exist, the role of this adipokine in obesity and insulin resistance has yet to be clearly defined.

### 5.7. Chemerin

Recently, chemerin (retinoic acid receptor responder 2, tazarotene-induced gene 2) was found to be highly expressed in adipose tissue and liver [165]. Chemerin is an agonist of the orphan G-protein coupled receptor chemokine-like receptor 1 (CMKLR1, ChemR23) [166] that is expressed by cells of the innate immune system [167]. Therefore, chemerin might be further evidence of a link between obesity and inflammation. Chemerin is secreted as an inactive precursor, and then activated through proteolytic cleavage by serine proteases of the coagulation, fibrinolytic and inflammatory cascades. Chemerin appears to be a novel and promising adipokine, and in several recent studies, human chemerin plasma levels have been shown to have a significant association with the BMI, inflammation, and metabolic syndrome [168–170].

Platelets have been found to be a rich cellular source of chemerin. In some pathological conditions, chemerin is activated and then released, which leads to the elevation of blood chemerin levels [171]. Recent studies have shown that both adipocytes [172] and fibroblast cells [173] can produce chemerin. Chemerin has also been measured in a number of human inflammatory exudates, including ascitic fluids from human ovary cancer and liver cancer, as well as synovial fluids from arthritic patients [174]. Angiotensin-converting enzyme (ACE) may be responsible for the activation of prochemerin. If so, as has been shown in vitro, this effect should be able to be blocked by an ACE inhibitor such as captopril [175]. However, further studies will be necessary to clarify this potential mechanism in vivo. There is also growing evidence that the bioactivity of chemerin is closely regulated by proteolytic cleavage in the C-terminal region, which may control its maximal chemotactic or anti-inflammatory effects [176]. While the primary amino acid sequences indicate that chemerin is structurally distinct from the CXC and CC chemokines, it functions exactly like a chemokine and can induce leukocyte migration and intracellular calcium mobilization. Chemerin also exerts potent anti-inflammatory effects on activated macrophages, which express the chemerin receptor CMKLR1 (chemokine-like receptor-1) in a cysteine protease-dependent manner [177].

Chemerin is a newly described adipokine with effects on adipocyte differentiation and metabolism in vitro [165]. In rodents, there is conflicting data with regard to the association of chemerin with obesity and diabetes. While there is a decreased chemerin expression in the adipose tissue of db/db mice as compared with controls [178], chemerin expression is significantly higher in the adipose tissue of impaired glucose tolerant and diabetic Psammomys obesus as compared with normal glucose-tolerant sand rats [179]. It has also been demonstrated that chemerin or chemerin receptor knockdown impairs the differentiation of 3T3-L1 cells into adipocytes, reduces the expression of adipocyte genes involved in glucose and lipid homeostasis, including adiponectin and leptin, and alters the metabolic functions in mature adipocytes [170]. In humans, no significant differences were noted for the chemerin levels between subjects with type 2 diabetes and normal controls. However, in normal glucose-tolerant subjects, chemerin levels were significantly associated with BMI, triglycerides, and blood pressure [179]. Plasma chemerin levels in normal subjects are also significantly associated with BMI, circulating triglycerides, and blood pressure, suggesting a strong relationship of this protein with obesity-associated complications [179].

It is possible that visceral fat may potentially contribute to the chronic inflammation that is observed in obese individuals. However, only a few studies have investigated the adipokine concentrations in the portal circulation [180]. In order to be able to determine the physiological role of chemerin in the glucose metabolism, and to identify chemerin's target tissues as well as relevant signal transduction pathways, further studies will need to be undertaken.

### 5.8. Omentin, Apelin, Vaspin, and Retinol-Binding Protein 4 (RBP4)

Omentin, which was originally referred to as intelectin and first found in the intestinal Paneth cells, has a predicted molecular weight of 33 kDa [181]. Omentin is a fat depot-specific secretory protein synthesized by the visceral stromal vascular cells, but not the adipocytes. It has also been found in the human lung, intestine, and heart [182] and is strongly expressed in the human ovaries and placenta [183]. This new adipokine is codified by two genes (1 and 2) and is highly and selectively expressed in the visceral adipose tissue. In obesity, omentin 1 plasma levels and the adipose tissue gene expression are decreased, and there is a positive correlation with the plasma adiponectin and high-density lipoprotein. These levels were negatively correlated with waist circumference, BMI, and insulin resistance [184, 185]. Administration of glucose and insulin to human omental adipose tissue explants resulted in a dose-dependent reduction of the omentin-1 expression. Furthermore, prolonged insulin-glucose infusion in healthy individuals resulted in significantly decreased plasma omentin-1 levels [186]. Recombinant omentin enhances the uptake of glucose in isolated adipocytes and dramatically increases the insulin induction of Akt/PKB phosphorylation [182]. However, further studies need to be undertaken, as the physiological role of omentin in glucose metabolism along with omentin's target tissues, receptor, and the relevant signal transduction pathways have yet to be determined.

Apelin is a bioactive peptide that is produced by adipocytes, vascular stromal cells, the heart, and the cardiovascular system [187]. In humans, both obesity and insulin significantly elevate the plasma levels of apelin and this peptide appears to act as a circulating and paracrine hormone [187]. The gene that encodes the apelin, receptor shares the greatest sequence identity with the angiotensin AT1 receptor [187]. In experimental animal models of heart failure, the cardiac apelin system is down-regulated by angiotensin II, while restoration is achieved after treatment with an angiotensin type 1 receptor blocker [188]. In the cardiovascular tissues of rats, apelin production is up-regulated by hypoxia [189] and ischemic cardiomyopathy [190], which perhaps may be a compensatory mechanism. In spontaneously hypertensive rats, exercise training has also been shown to up-regulate the apelin production [191]. Apelin has a positive hemodynamic effect, as it acts an inotrope in both normal and failing rat hearts and in isolated cardiomyocytes [192, 193]. Apelin may be able to regulate insulin resistance by facilitating the expression of brown adipose tissue uncoupling proteins and by altering adiponectin levels [194]. Decreased plasma apelin levels have been observed in patients with lone atrial fibrillation [195] and chronic heart failure [196]. Cardiac resynchronization therapy has been used to treat these patients successfully, with increases in the apelin levels observed after initiation of the therapy [197].

Vaspin is a member of the serine protease inhibitor family. This adipocytokine has been isolated from the visceral adipose tissue of Otsuka Long-Evans Tokushima Fatty (OLETF) rats that are at an age when the body weight and hyperinsulinemia has peaked [198]. OLETF rats are commonly used as a model of human type 2 diabetes. This model also shares common components of the human metabolic syndrome, including abdominal obesity, insulin resistance, hypertension, and dyslipidemia [199]. Vaspin production decreased at the same time the diabetes worsened and body weight fell in the untreated OLETF rats. However, when the animals were treated with insulin or pioglitazone, serum vaspin levels were maintained [198]. This suggests that the up-regulation of vaspin may have a defensive action against insulin resistance. Human vaspin mRNA has been reported to be expressed in the visceral and subcutaneous adipose tissue. In addition, it has been shown to be regulated in a fat-depot specific manner, and to be associated with obesity and parameters of insulin resistance [200, 201]. It has also been reported that elevated vaspin serum concentrations are correlated with obesity and impaired insulin sensitivity, whereas type 2 diabetes appears to abrogate this correlation [202, 203]. Vaspin expression decreases in conjunction with a worsening of the diabetes and a body weight loss. These studies indicated that vaspin might play a causative role in the development of obesity and metabolic disorders or, at least, be a biomarker for these diseases. In order to clarify these potential mechanisms, further investigation using more sophisticated methods will need to be undertaken.

Using the adipose-specific Glut4 knockout (adipose-Glut4(−/−)) mice model, retinol-binding protein 4 (RBP4) has been identified as a highly expressed circulating adipokine that causes insulin resistance when it is overexpressed or injected into mice [204]. In the circulation, RBP4 is bound to transthyretin, which causes decreases in the RBP4 renal clearance. In ob/ob mice, there was a 4-fold increase in transthyretin plasma levels as compared to lean mice or diet-induced obese mice [205]. A large number of subsequent studies confirmed there was an association between increases in the circulating RBP4 levels and various aspects of adiposity [206], insulin resistance [207, 208], diabetes mellitus [209], and metabolic syndrome [210, 211]. However, there are also other studies that have been unable to establish these associations [212, 213]. The reason for this discrepancy may be explained in part by the different methods that were used to measure the RBP4 and the different populations employed in these various studies. In some very recent studies, it has been reported that increased plasma RBP4 levels are associated with inflammatory cardiomyopathy [214] and cerebral infarction [215]. Therefore, at the current time, whether RBP4 functions as an adipokine in humans and exerts metabolic effects on glucose metabolism remains uncertain. Further studies will need to be performed in order to clarify RBP4's exact role in humans.

## 6. Conclusions

The worldwide incidence of obesity has markedly increased during recent decades. Obesity and associated disorders now constitute a serious threat to the current and future health of all populations on earth. Obesity represents a major risk factor for diseases including CVD,,atherosclerosis and diabetes, in which inflammation acts as a major driver in the pathogenesis. Both adipocytes and macrophages within fat tissue secrete numerous cytokines that may contribute to the characteristic pathophysiological changes. By expanding our knowledge on inflammation and the link between obesity and CVD, this should make it possible to improve our understanding of the pathophysiology of obesity.
